# Varicella Death of an Unvaccinated, Previously Healthy Adolescent — Ohio, 2009

**Published:** 2013-04-12

**Authors:** Jeremy Budd, Brian Fowler, Alise Brown, Sandy Swann, Jessica Leung, Mona Marin

**Affiliations:** Bur of Infectious Diseases, Ohio Dept of Health; Trumbull County Health Dept, Ohio; Div of Viral Diseases, National Center for Immunization and Respiratory Diseases, CDC

Varicella usually is a self-limited disease but sometimes can result in severe complications and death. Although infants, adults, and immunocompromised persons are at increased risk for severe disease, before varicella vaccine was introduced in 1995, the majority of hospitalizations and deaths from varicella occurred among healthy persons aged <20 years (1). Introduction of varicella vaccine has substantially decreased varicella incidence, hospitalizations, and deaths in the United States (2). This report describes a varicella death in an unvaccinated, previously healthy adolescent aged 15 years. In April 2012, as part of the routine review of vital statistics records, the Ohio Department of Health identified a 2009 death with the *International Classification of Diseases, 10th Revision* code for varicella as the underlying cause. Because varicella deaths are nationally reportable, the Ohio Department of Health conducted an investigation to validate that the coding was accurate. Investigators learned that, on March 12, 2009, the adolescent girl was admitted to a hospital with a 3-day history of a rash consistent with varicella and a 1-day history of fever and shortness of breath. The patient was started on intravenous acyclovir (on day 4 of illness) and broad-spectrum antibiotics and antifungals, but she died 3 weeks later. The case underscores the importance of varicella vaccination, including catch-up vaccination of older children and adolescents, to prevent varicella and its serious complications.

On admission, the patient had a fever of 101.1°F (38.4°C), dyspnea, facial edema, generalized petechial rash, and hypotension; she received a diagnosis of septic shock. She was awake and alert, and noninvasive mechanical ventilation was implemented during the first 6 hours of admission. However, her respiratory function continued to deteriorate with increasingly labored breathing, requiring invasive mechanical ventilation.

The patient’s laboratory results at admission indicated thrombocytopenia (platelet count: 30,000/*μ*L; normal: 140,000–400,000/*μ*L) and leukopenia (white blood cell count: 1,400/*μ*L; normal: 3,800–10,600/*μ*L); blood cultures were negative. Vesicular fluid from a skin specimen collected on March 14 was positive for varicella-zoster virus (VZV) by direct fluorescent antibody test. Over the course of hospitalization, the patient developed pneumonia complicated by acute respiratory distress syndrome, pancytopenia, multi-organ dysfunction, health-care–acquired bacterial colonization and infection (including respiratory tract colonization with *Enterobacter cloacae* and urinary tract infection with *Pseudomonas aeruginosa*), and sepsis (blood cultures on hospital days 19 and 20 were positive for *Stenotrophomonas maltophilia)*. Other blood cultures were negative, but they had been collected while she was on antibiotics.

Multiple chest radiographs showed diffuse, tiny nodules in the lung parenchyma consistent with alveolar consolidation. A computed tomography scan did not find any intracranial lesions, and electroencephalography ruled out any subclinical seizures. In addition to initial treatment with intravenous acyclovir, the patient’s treatment included ciprofloxacin, meropenem, trimethoprim-sulfamethoxazole, ticarcillin-clavulanate, and tigecycline. During her last week in the hospital, her respiratory function deteriorated progressively, requiring high levels of pressure and oxygen. On hospital day 21, she died.

The source of the patient’s VZV exposure remains unknown. She had previously received 4 doses of diphtheria-tetanus-pertussis vaccine; 1 dose of *Haemophilus influenzae* type b vaccine; and 2 doses of measles-mumps-rubella vaccine, but lived in a community with low rates of varicella vaccination. She did not have any known underlying medical conditions. An aspirate of bone marrow obtained during her hospitalization showed no evidence of leukemia.

## Editorial Note

Varicella vaccine is highly effective (>95%) in preventing severe varicella and deaths (2). VZV infection has the potential, even among healthy persons, to cause severe complications, including secondary bacterial infection and sepsis, pneumonia, encephalitis, cerebellar ataxia, and thrombocytopenia; these complications can occur within a few days of rash onset (1,3).

The Advisory Committee on Immunization Practices recommends routine administration of the first dose of varicella vaccine at age 12–15 months and the second dose at age 4–6 years. Catch-up vaccination also is recommended. Unvaccinated persons who do not have evidence of immunity to varicella* should receive 2 doses of varicella vaccine at appropriate intervals, and those who have received 1 dose previously should receive a second dose (2).

Before varicella vaccination was included in routine childhood immunization, approximately 11,000 varicella-related hospitalizations and 100–150 deaths were reported annually in the United States (2). Implementation of the varicella vaccination program in the United States has led to declines of >95% in varicella-related illnesses, hospitalizations, and deaths in populations that received routine vaccination. However, of 24,488 varicella-related hospitalizations during 2000–2006, a total of 17,142 (70%) were among healthy persons with no contraindications for vaccination (4). Among 112 varicella-related deaths during 2002–2007, a total of 100 (89%) were among persons with no high-risk preexisting conditions, such as cancer, immunodeficiency, or pregnancy (5).

The case described in this report can serve as a reminder of the importance of catch-up vaccination of older children and adolescents (2) to prevent varicella and its serious complications later in life when disease can be more severe. Approaches that are used to implement catch-up vaccination include school-entry vaccination requirements and routine health-care provider visits. Ohio currently has a 2-dose varicella vaccination requirement for admission to kindergarten through 2nd grades, and a 1-dose requirement for admission to 3rd–6th grades. However, when the patient aged 15 years contracted varicella, no varicella vaccination school-entry requirements covered her grade. With continued implementation of the 2-dose requirement in Ohio, an additional grade will be added each school year so that, by 2022, the requirement will cover all grades; religious and medical exemptions will continue to be allowed. To cover cohorts of students enrolled in school before elementary school requirements took effect, implementation of varicella vaccination entry requirements for students entering middle school, high school, and college should be considered (2). Routine health-care provider visits, including the recommended visit at age 11–12 years, also provide an opportunity to evaluate vaccination status and administer recommended vaccinations (6).

Exposure to VZV can occur when persons are exposed to patients with varicella (chickenpox) or herpes zoster (shingles). Unvaccinated children, adolescents, and adults are at risk for acquiring varicella; severe varicella can develop among healthy persons, and which patients might develop an especially severe course often is unpredictable at disease onset. Health-care providers should remind parents about vaccination during routine visits for children and adolescents, and parents should be informed of the risks, including potentially severe complications, from vaccine-preventable diseases. Resources for discussions with parents regarding vaccination are available.† Adult patients who have no evidence of varicella immunity should be offered varicella vaccine. For otherwise healthy persons aged >12 years who develop varicella, oral acyclovir is recommended. Treatment should be initiated as soon as possible, ideally within the first 24 hours (7). Intravenous acyclovir therapy is recommended for immunocompromised patients, and also in cases with serious, viral-mediated complications (7).

What is already known on this topic?

Although varicella usually is a self-limited disease, it can lead to severe complications and death, even among persons without underlying conditions that put them at increased risk for severe disease. The varicella vaccine is highly effective in preventing severe varicella and death.

What is added by this report?

This report describes a varicella-related death that occurred in an unvaccinated, previously healthy adolescent aged 15 years. The case described in this report can serve as a reminder of the importance of catch-up vaccination of older children and adolescents to prevent varicella and its serious complications later in life when disease can be more severe. The case underscores the fact that severe complications of varicella and death can occur among persons without high-risk conditions for severe varicella.

What are the implications for public health practice?

Severe varicella can develop among unvaccinated healthy persons, and which patients might develop an especially severe course often is unpredictable. Persons without evidence of immunity to varicella should receive 2 doses of varicella vaccine, or a second dose if they have received only 1 dose, to prevent varicella and its severe complications.

## Footnotes

* Evidence of immunity to varicella includes any of the following: 1) documentation of age-appropriate vaccination with varicella vaccine, 2) laboratory evidence of immunity or laboratory confirmation of disease, 3) birth in the United States before 1980, or 4) diagnosis or verification by a health-care provider of a history of varicella or herpes zoster disease.

† Available at http://www.cdc.gov/vaccines/hcp/patient-ed/conversations/index.html.

## References

[1] Arvin AM (1996). Varicella-zoster virus. Clin Microbiol Rev.

[2] CDC (2007). Prevention of varicella: recommendations of the Advisory Committee on Immunization Practices (ACIP). MMWR.

[3] Jackson MA, Burry VF, Olson LC (1992). Complications of varicella requiring hospitalization in previously healthy children. Pediatr Infect Dis J.

[4] Lopez AS, Zhang J, Brown C, Bialek S (2011). Varicella-related hospitalizations in the United States, 2000–2006: the 1-dose varicella vaccination era. Pediatrics.

[5] Marin M, Zhang JX, Seward JF (2011). Near elimination of varicella deaths in the US after implementation of the vaccination program. Pediatrics.

[6] CDC (1996). Immunization of adolescents: recommendations of the Advisory Committee on Immunization Practices, the American Academy of Pediatrics, the American Academy of Family Physicians, and the American Medical Association. MMWR.

[7] Pickering LK, American Academy of Pediatrics (2012). Varicella-zoster infections. Red book: 2012 report of the Committee on Infectious Diseases.

